# Survey of Nursery Errors in Healthcare Centers, Isfahan, Iran

**DOI:** 10.5539/gjhs.v8n3p43

**Published:** 2015-06-25

**Authors:** Ali Ayoubian, Mansooreh Habibi, Pouria Yazdian, Hossein Bagherian-Mahmoodabadi, Peyman Arasteh, Tannaz Eghbali, Tohid Emami Meybodi

**Affiliations:** 1 Health Research Center, Baqiyatallah University of Medical Sciences, Tehran, Iran; 2 Mazandaran University of Medical Sciences, Sari, Iran; 3 Student Research Committee, Shahid Sadughi University of Medical Sciences, Yazd, Iran; 4 Department of Health Services Management, Isfahan University of Medical Sciences, Isfahan, Iran; 5 Non Communicable Diseases Research Center, Fasa University of Medical Sciences, Fasa, Iran; 6 Student Research Committee, Shiraz University of Medical Sciences, Shiraz, Iran; 7 Shahid Sadoughi University of Medical Sciences, Yazd, Iran

**Keywords:** nursery mistakes, nurse, delivery of health care

## Abstract

**Background & Aim:**

Nurse's mistakes usually have a strong effect on the patients trust and satisfaction in the health services systems, and it can also lead to stress and moral contradicts among nurses. This study has aimed to survey the rate of nurses’ mistakes, according to documents in the Isfahan Province during 2007-2012.

**Methods:**

The study was a descriptive cross-sectional study. The sample population consisted of all complaints concerning nursing services provided in hospitals, private clinics and other health service centers between 2007 and 2012, submitted to the Forensic Medicine Commission Office, in Isfahan. The data were collected by a cheklist and analyzed using SPSS version 16.0 software.

**Results:**

Out of 708 complaints, 70 (9.8%) cases were related to nurses. Twenty-four cases led to awards. The age range of nurses was 35-40 (25.7%). Out of 70 nurses with a record, 75% (53 people) were female and the rest were male. Sixty four nurses (91.4%) were working in hospitals. Negligence was the first basis of the court rulings (16 cases out of 24). Nurses’ recklessness in providing services was due to their convictions among 66.7% of the cases

**Conclusion:**

Although efforts to reduce and control nurses’ faults and mistakes depends on using a system for studying and removing the factors which lead to faults, human error is inevitable in every occupation and a 100% accurate operation is unreachable.

## 1. Background

Human beings always make mistakes. This is also true for the health care staff and authorities. Regardless of their skills, experiences, and commitments, the nurses may and will make mistakes. There are thousands of cases of injuries or even deaths recorded in the USA as a result of physicians and nurses’ faults and mistakes. More than 2000 deaths are recorded due to nursing mistakes in the USA each decade. Nursing mistakes may bring dire consequences to the patients (Anderson & Webster, 2001).

The international council of nurses declared that developing the quality of services by nurses is necessary to improve the patients’ health and all nurses are responsible to preserve the patients’ security in all aspects. This includes making the patients and personnel informed about the possibility of faults, methods for reducing the rate of mistakes, supporting patients’ security and reporting any cases to the authorities (Johnstone & Kanitsaki, 2006).

The performance of the nurses is usually questioned more than any other staff in health services (Cho et al., 2009) -particularly by physicians- concerning the role of nurse as the most important member of developing the quality of health services. Similar to the management of the medication, nurses also have the responsibility for the patients’ security, consequently, they might be accused of wide ranges of issues (Joolaee, Hajibabaee, Peyrovi, Haghani, & Bahrani, 2011).

Balas et al. showed that 30% of the nurses participating in their study had made a mistake at least once (Balas, Scott, & Rogers, 2004). Several studies have been carried out on estimating the nursing and medical mistakes. A study on physicians’ activity in Harvard University showed that more than 70% of the unwanted incidents had been due to the poor performance of the medical staff and more than 90% of them were preventable (Burroughs et al., 2005).

In accordance with the studies by the American Medical Association, at least 100 patients die because of the faults in the health services and 92% of the staff believe that nurses are largely to blame (Frith, 2013).

Medical mistakes are usually brought to the court under the titles of 1- Skill-based errors; 2- Rule-based errors; and 3- Knowledge-based errors. However, the errors by nature are usually unintentional. Studies in the European countries showed that nursing mistakes are sustained by 18-28% of the patients (Reason, 2000). It is notable that informing the public about patients’ bill of right draws the patients’ attention to the health services in hospitals. In this case many patients will take legal action against health staff neglecting health care standards (Ayoubian, Mahmoodabadi, & Dehaghi, 2013).

The medical faults in Iran are proposed under the topic of medical faults and refer to the responsibility of the providers of services for the damages and losses occurred during treatment (Zeraatchi, Talebian, Nejati, & Dashti-Khavidaki, 2013). The complaints about medical faults usually fall into 4 groups: carelessness, incautious, lack of proficiency, and lack of observance of governmental derivatives. However, the faults generally are unintentional. On one hand, there are no categorized data and information regarding the reasons of the faults and types of them and the available information are about the occurrence of the faults in Iran.

This is not because of low rates of faults in Iran, but because of lack of accurate recording and registration systems and research studies in this field. On the other hand, the increase of referred files and the patients’ complaints against physicians and nurses to medical system administrations and courts depicts the existence and the increase of faults in this group of people (Joolaee et al., 2011).

## 2. Objective

The occurrence of such faults leads to destroy trust and, consequently, the dissatisfaction of patients. In this case it can be said that health services can bring about stress and moral conflicts for the nurses and health service staff. Therefore, this study has aimed to demonstrate the rate of mistakes committed by nurses between the years 2007-2012.

## 3. Methods

The study is a descriptive cross sectional work. The study population was comprised of all the submitted complaints to Isfahan Province Forensic Medicine Commission Office regarding nursing services provided in hospitals, private clinics and other health services centers between 2007 and 2012. The complaints had been made from April 2007 to March 2012 (n=708). Owing to the paucity of the sample size, census sampling was used. Totally, there were 70 complaints regarding the nursing services (9.8%) and all these cases were examined in this work.

The participation criteria were i) the complaints must be about poor nursing services provided in hospitals, clinics, and other health service centers; ii) the complaints must be submitted between April 2005 and March 2010; and iii) the complaints must be examined and finalized by the commission. The exclusion criterion was that the complaint was not about poor services by the nursing staff.

Data were collected using a cheklist which was designed based on age, gender, education level, subject of complaint, result of the case, and place of service. The validity of the check list was confirmed by the experts and scholars. The study was carried out in the archive department of Isfahan Province Forensic Medicine Organization in spring 2012. During this study, all data collection processes were done after securing the required permissions, the researchers made their best not to interrupt the normal work process of the department; and the confidentiality of the information was observed.

Manual work procedure in the department (no computerized system was available) and limited time to examine the files and confidentiality of the information were some of the limitations of the study. Data analysis was carried by using SPSS version 16.0 software.

## 4. Results

As listed in Table 1, the largest group of cases was filed against staff in the age ranges of 35 to 40 (25.7%). Next in line were the age groups of 30 to 35 and 20 to30 with 15 and 17 files respectively.

**Table 1:** Frequency of complaints against nurses, by the age of nurses

Age	N	%
**20-25**	6	8.6
**25-30**	15	21.4
**30-35**	17	24.3
**35-40**	18	25.7
**40-45**	7	10
**45-50**	4	5.7
**50-55**	3	4.3

**Total**	**70**	**100**

Women constituted 75.7% (53 out of 70) of the sample group. Out of the 70 nurses in the study, 24 cases led to conviction and 46 to acquittal ruling.

Table 2 lists the frequency of place service provision. Among nurses studied on, 64 (91.4%) worked in hospitals. None of the cases occurred in private clinics.

**Table 2:** Frequency of complaints against nurses by nurses’ work place

Location of Service	N	%
**Hospital**	64	91.5
**Private Clinic**	1	1.4
**Home Care**	4	5.7
**Other**	1	1.4

**Total**	**70**	**100**

Incautiousness was one of the most important bases of conviction of nurses in 66.7% (16 out of 24 cases). Lack of observation and governmental instruction 16.7%, carelessness 12.5%, and low proficiency 4.1% were next on the line.

The faults had occurred because of reasons such as carelessness, administrating without the permission of the supervisor, loss of the count of gauzes and leaving one in the patient's stomach, immature child delivery, failing to take care of mental patients, using wrong gauzes, failing to inform physicians about patients’ conditions, providing services out of professional competency, unpermitted hospitalization and default in the attendance of the patient.

The findings show that on average the court ruling was one adult blood-money in 23% of the cases, half of adult blood-money was the most common ruling and 1.5% of the adult blood-money was the minimum conviction. Also among 70 complaints against the nurses that have been studied, 24 cases of them (34.3%) led to convictions and 46 cases (65.7%) have led to exoneration.

Table 3 suggests that in 50% of the cases (12 out 24) conviction was 1-10% of an adult blood money. None of the cases resulted in more than 50% of the adult blood-money.

**Table 3:** Frequency of convicted nurses by an adult blood money

Penalty Fee	N	%
**1-10**	12	50
**11-20**	1	4.2
**21-30**	3	12.5
**31-40**	1	4.2
**41-50**	7	29.1
**Total**	**24**	**100**

Table 4 shows that nurses’ recklessness in providing services was due to their convictions among 66.7% of the cases.

**Table 4:** Frequency of the cause of nurses’ conviction

Cause	N	%
**Imprudence**	3	12.5
**Recklessness**	16	66.7
**Lack of Skill**	1	4.1
**Lack of Comply with Government System**	4	16.7
**Total**	**24**	**100**

## 5. Discussion

Results show that out of the 708 complaint cases 70 cases (9.8%) were against nurses. In a study by Frouzesh, the figure was reported 2%, which is a considerable difference in compared to our findings (Foruzesh, Ghorbani, Vosugh, & Mohammadi, 2011). Concerning the 70 cases against nurses, 24 (34.3%) leaded to conviction (44.3%) and 46 (65.7%) to acquittal. Negligence, breach of instructions, remiss, and lack of enough skill were the main bases of the rulings.

Prohibited activities in provision of medical services are a common case in Iranian hospitals. It is a common case where the nurse provides medical service out of their competency, which ends up with negative side effects on the patients and file of complaints. Regarding other ranks of nursing such health staff and health assistants, there are many cases where they are instructed by nurses to provide services that they do not have the competency for. There are many cases in courts filled by those sustained unwanted side effects by such services (Iyer, 2002).

Nelson et al. reported that handing over non-health care services such as preparing specimens, computer stuff, secretary services, over works and tiredness, assignment of physicians’ tasks, bad penmanship of physicians, lack of pharmacological knowledge, pressure for more services, and employment of students instead of qualified personnel, improper physician shifts, lack of access to pharmacologists for consultation are of the main causes of faults of nursing services (Nelson, Evans, Samore, & Gardner, 2005).

On the other hand, reports by studies in other countries showed that human factors are effective in nursing services (Nelson et al., 2005). Consistently, our results showed that negligence is the main cause of faults in nursing services. However, this is inconsistent with the results obtained by Nelson.

Furthermore, the results showed that nurses in hospitals under the supervision of the University of Medical Sciences and Social Security Organization had the maximum number of complaints. This is not surprising taking into account the extension of the services provided by these two groups of hospitals. Therefore, this unconfirmed result seems reasonable enough.

The majority of the nurses were women, which is expectable concerning the fact that the majority of the nurses are women. The results showed that there are several factors to blame as to preparing the ground of making nursing mistakes. The first way to cut these mistakes is to spot the causes (Kingston, Evans, Smith, & Berry, 2004).

In addition, creating an environment where all nursing staff declare their mistakes to their colleagues, managers, and medical team and creating opportunities to compensate the mistakes is another way to deal with medical mistakes and faults. The authorities need to collect information of the types of mistakes, circumstances (work shift, staffing, personal characteristics, and soon) and use the information to deal with the causes of mistakes (Johnstone & Kanitsaki, 2006).

It is expectable that nurses are the first group of staff who are blamed for bad services, however, what may help the staff under hard working condition is to inform them about the rules and regulations (Green, 2004).

## 6. Conclusion

Human mistakes are intrinsic to any profession and having a system which is free of fault is not achievable. However, the implementation of a systematic approach to deal with the causes of faults and removing them are the keys to control the rate of staff faults.

The results showed that the main cause of the faults done by nurses is negligence. To deal with this issue and mainly negligence-based mistakes, it is recommended to hold periodical recalling courses for the nurses, to supply required qualified nursing staff for health centers and to implement an effective system to distribute patients to the capacity of the health services. New approaches to keep the nurses’ update (based on recent publications, books, journals, etc.) must be developed. Work mistakes take place either due to lack of skills or negligence.
